# Update on the Health Services Research Doctoral Core Competencies

**DOI:** 10.1111/1475-6773.12851

**Published:** 2018-03-13

**Authors:** James F. Burgess, Nir Menachemi, Matthew L. Maciejewski

**Affiliations:** ^1^ Center for Health Services Research in Primary Care Durham VA Medical Center Durham NC; ^2^ Department of Population Health Sciences Duke University School of Medicine Durham NC; ^3^ Center for Healthcare Organization and Implementation Research VA Boston Healthcare System Boston MA; ^4^ Boston University School of Public Health Boston MA; ^5^ Indiana University Richard M. Fairbanks School of Public Health Indianapolis IN; ^6^ Regenstrief Institute Center for Biomedical Informatics Indianapolis IN

**Keywords:** Health services research, training, competencies, doctoral core competencies

## Abstract

**Objective:**

To present revised core competencies for doctoral programs in health services research (HSR), modalities to deliver these competencies, and suggested methods for assessing mastery of these competencies.

**Data Sources and Data Collection:**

Core competencies were originally developed in 2005, updated (but unpublished) in 2008, modestly updated for a 2016 HSR workforce conference, and revised based on feedback from attendees. Additional feedback was obtained from doctoral program directors, employer/workforce experts and attendees of presentation on these competencies at the AcademyHealth's June 2017 Annual Research Meeting.

**Principal Findings:**

The current version (V2.1) competencies include the ethical conduct of research, conceptual models, development of research questions, study designs, data measurement and collection methods, statistical methods for analyzing data, professional collaboration, and knowledge dissemination. These competencies represent a core that defines what HSR researchers should master in order to address the complexities of microsystem to macro‐system research that HSR entails. There are opportunities to conduct formal evaluation of newer delivery modalities (e.g., flipped classrooms) and to integrate new Learning Health System Researcher Core Competencies, developed by AHRQ, into the HSR core competencies.

**Conclusions:**

Core competencies in HSR are a continually evolving work in progress because new research questions arise, new methods are developed, and the trans‐disciplinary nature of the field leads to new multidisciplinary and team building needs.

Health Services Research (HSR) remains a complex multidisciplinary field with a history of more than 50 years (Zinn et al. 2017) that is defined by its investigation of complex health policy and practice questions affecting the health and health care of individuals and populations. HSR is often done as team science comprised of social scientists, clinical experts in medicine, nursing, or other allied health fields, and members with experience on the frontlines of the clinical, managerial, or policy issue under investigation. To formally represent the substantive training provided in HSR doctoral programs and to clearly distinguish HSR training from other postgraduate opportunities for students and employers, a list of core competencies were first developed in a 2005 Agency for Healthcare Research and Quality (AHRQ)–funded conference. Version 1 (V1) of the HSR Doctoral Core Competencies (Forrest et al. 2009) was generated at this conference through consensus and productive communication across disciplines with different research vernaculars and traditions.

Consensus on the V1 core competences was facilitated by the framework provided by the definition of HSR developed by Lohr and Steinwachs (2002):

Health services research is the multidisciplinary field of scientific investigation that studies how social factors, financing systems, organizational structures and processes, health technologies, and personal behaviors affect access to health care, the quality and cost of health care, and ultimately our health and well‐being. Its research domains are individuals, families, organizations, institutions, communities, and populations.

Shortly upon completion of V1 competencies, AHRQ held a 2008 conference that led to further evolution of the core competencies in an unpublished V2. (Martin 2008) With the lack of widespread dissemination of the V2 core competencies, an opportunity to revisit them became possible when AHRQ, Robert Wood Johnson Foundation, and the Patient Centered Outcomes Research Institute (PCORI) funded a November 2016 conference, hosted by AcademyHealth, to understand the future needs of the HSR workforce.

An update to the HSR competencies was deemed appropriate because HSR as actively practiced today has evolved to account for important changes in health policy, care delivery models, and data sources that prompt new research questions. For example, there are new research questions regarding the effectiveness of value‐based purchasing, interventions to reduce low‐value care, and the patient and health system benefits of Accountable Care Organizations and bundled payments. Moreover, there is increasing availability of EHR data, greater recognition of the importance of mixed‐methods studies, and growing interest in cutting‐edge methods (e.g., machine learning) for generating knowledge from “big data.” Finally, the workforce with HSR training has grown (Frogner 2018) since the 2005 conference when the V1 competencies were first developed, particularly in the private sector, where health delivery systems, data analytics companies, and policy research organizations have great demand for doctorally trained HSR professionals (Rich and Collins 2018).

It is critical to revisit the HSR core competencies to be sure that the field is anticipating the current and future needs of these employers. For employers, revised core competencies can signal the cognitive and technical skills that HSR trainees will have upon graduation and potentially indicate gaps between these competencies and essential skills and knowledge desired by employers. For doctoral programs, revised core competencies can help them refine existing course offerings or consider new courses to address evolutions in data, methods, and health delivery models. For prospective students, revised core competencies can aid them in choosing the doctoral program that most suits their professional interests and aspirations.

The revised competencies are intended to be a resource for stakeholders to inform skill development that begins in training programs and continues as lifelong learning throughout one's career in the HSR workforce of the future. In this paper, we present two tables that were developed for V2 that summarize some potential ways to deliver these competencies and to assess mastery of these competencies. Additionally, we present a typology of health services researcher types that can help training programs prioritize competencies as they evolve over time to address new issues in HSR.

## Methods

In preparation for the November 2016 conference on the HSR Workforce, the lead author (JB) was tasked with developing a white paper on updated HSR competencies where he presented the unpublished V2 competencies to conference participants for discussion and feedback. To develop the white paper, the lead author reviewed V1 core competencies originally developed in 2005 (that were published in 2009) and the V2 competencies that were developed for the 2008 conference. Conference participants included academic, governmental, and private sector stakeholders of health services researchers, including educators, students, and employers. Additional feedback was solicited from doctoral program directors and other HSR workforce experts that participated in AcademyHealth's HSR Learning Consortium. This feedback led to modest revisions by the lead author, which were then presented (by NM) at AcademyHealth's June 2017 Annual Research Meeting in New Orleans. Additional feedback was received from session participants including two invited discussants.

The current core competencies, henceforth referred to as V2.1, represent the authors’ attempt to synthesize and incorporate the solicited feedback generated in the above activities. Importantly, after the untimely passing of the first author, the other authors were invited to take responsibility for the submitted version of this manuscript. Having not been part of the author team until that point, we held weekly conference calls in August and September 2017 to piece together the record and feedback received in order to complete the current manuscript. Since the V2.1 competencies were originally developed by the lead author alone and then modestly revised by the other authors following the AcademyHealth presentation, the V2.1 competencies presented below are entirely based on the authors’ opinions. In the course of the revision process, the second author added a typology of health services researchers that was not part of the original submission, which was based on his experience with many multidisciplinary teams.

Given this process, some of the materials presented herein hew closely to the V2 competencies generated during a 2008 AHRQ‐funded conference. These competencies were originally developed for a United States audience, and the same perspective is applied to these V2 competencies. However, the competencies, delivery modalities, and assessment methods likely generalize to doctoral programs outside the United States.

## Results

### Core Competencies

The original 14 core competencies outlined in V1 were reduced to 11 competencies in V2, which were retained with edits in V2.1 (Table 1). The number of competencies were reduced because one competency (*Apply in‐depth disciplinary knowledge and skills relevant to HSR*) was subsumed under others; one new competency merged two separate competencies about primary and secondary data collection; and one additional new competency merged two competencies regarding ethical and protocolized conduct of research. The current V2.1 competencies include ethical conduct of research, conceptual models, the development of research questions, study designs, data measurement and collection methods, statistical methods for analyzing data, professional collaboration, and knowledge dissemination.

**Table 1:** Health Services Research Doctoral Core Competencies

No.	Label	Competency	Domain*Examples
1	Foundational knowledge	Acquire knowledge of the context of health and health care systems, institutions, actors, and environments	Health & biology Cost & financing of health care Organization of health care Health policy Access & use Quality of care Health/clinical informatics Outcomes & effectiveness Resource allocation Health behavior Social determinants of health Cross‐cultural & global perspectives
2	Conceptual knowledge	Apply or develop theoretical and conceptual models and skills relevant to health services research	*Variable depending on the discipline or interdisciplinary area of specialization:* Economics Epidemiology Psychology Sociology Anthropology Management and organizations Demography Operations research Political science Complexity theory Implementation science Multi‐disciplinary theory construction
3	Relevant and important HSR question development	Propose important research questions informed by structured evidence assessment, stakeholder positions, pertinent theoretical and conceptual models, and new data; and formulate solutions to health problems, practice, and policy	Scientific method and theory Proposal development Health policy applications Questions leading to solutions to health problems
4	Conceptual models and operational methods	Use or develop a conceptual model to specify study constructs for a health services research questions and develop variables that reliably and validly measure these constructs	Scientific method and theory Measurement and variables Concept models Conceptual framework development Theories and criteria for causal inference
5	Study designs	Recognize the strengths and weaknesses of study designs to appropriately address specific health services research questions	Longitudinal designs Survey research Qualitative designs Quantitative designs Mixed‐methods designs Intervention research Community‐based participatory research Evaluation research Quality improvement approaches
6	Data collection	Sample and collect primary health and health care data and/or assemble and manage existing data from public and private sources	Survey research Qualitative research Operations research Data acquisition Big data & data mining methods Database management Quality control Sampling Health/clinical informatics Population measures
7	Research study conduct management	Execute and document procedures that ensure the reproducibility of the science, the responsible use of resources, and the ethical treatment of research subjects	Responsible conduct of research Ethics Human subjects/IRBs HIPAA Contracts and DUAs Quantitative research Qualitative research Data acquisition Quality control & DMBs Research study management Organizations as subjects Health law and risk management
8	Data analysis	Demonstrate proficiency in the appropriate application of analytical techniques to evaluate HSR questions	Advanced HSR data analytic methods Economic evaluation, including CEA Statistical analyses Measure development Decision sciences Sampling Weights Qualitative analytic methods Quantitative analytic methods, including causal inference
9	Professional development	Work collaboratively in teams within disciplines, across disciplines, and/or with stakeholders	Teamwork Leadership Team management Conflict resolution Knowledge management Project management Stakeholder collaboration & involvement Negotiation Teaching & mentoring Cross‐cultural & global perspectives Lifelong learning Patient engagement skills integrating with research
10	Effective communication	Effectively communicate the process, findings, and implications of health services research through multiple modalities with stakeholders	Proposal development Dissemination Oral and written communication skills Marketing & persuasion techniques Cultural sensitivity Cross‐cultural & global perspectives
11	Knowledge transfer	Knowledge translation to policy and practice	Evidence‐based practice Evidence‐based policy Human factors research Complexity science Change management Operations research Health marketing Implementation science Translational research Tacit knowledge

Fundamental to mastery of these core competencies is an individual's ability to engage in critical thinking, which should be sharpened throughout the course of doctoral training and beyond. Each of the 11 competencies outlined in V2.1 has domain examples to illustrate some of the specific skills or methods germane to each competency. For example, the competency for *Posing Research Questions Informed by Stakeholders* could require an understanding of health policy applications, the development of compelling proposals, and a grounding in the scientific method for inquiry (just to name some example domains). Similarly, doctoral programs could facilitate student mastery of the competency of *Professional Development* through training in teamwork, leadership, project management, conflict resolution, stakeholder engagement, and/or other skills.

With the vast increase in “big data” from EHRs, wearable devices, all‐payer claims databases and other sources, opportunities exist to explore previously unaddressable research questions. These new data sources may also allow for improvements upon prior work through enhanced measurement of outcomes and confounders. The development of new care models (e.g., Accountable Care Organizations) and policy changes (e.g., bundled payment, value‐based purchasing) also introduces new research questions that health services researchers must be prepared to effectively address. Being able to propose clear research questions that address important issues is a critically important competency (#3 in Table 1) for all HSR practitioners, regardless of their disciplinary orientation or subject matter expertise. It is recognized that new data sources and new questions may require the application of new methods and that new data sources may enable new questions to be addressed.

In V1 and V2 of the core competencies, there was limited detail on the statistical methods that are central to the conduct of rigorous HSR beyond noting that they fell into qualitative, quantitative, and mixed‐methods categories. As a result, academic programs trying to determine what methodologies to include in didactic courses had no guidance or starting point regarding what HSR doctoral students should be able to perform themselves or consult with methodological experts who could assist them. It is beyond the scope of this manuscript to address this discussion in depth, but one of the challenges that the HSR field has faced imperfectly has been the use of quantitative, qualitative methods, and mixed methods, consistently and effectively to solve the complex problems we address through our work. It has been noted that qualitative methods have not been employed as well as could be possible (Devers 2011). There are other methodologic developments that doctoral programs could consider exposing students to in some capacity, including machine learning and advanced causal inference methods beyond the commonly taught difference‐in‐difference, propensity score, and instrumental variable methods. In addition, there is an increasing need for thoughtful selection of research methods when evaluating complex interventions in the dynamic environment of healthcare delivery or healthcare policy (Lamont et al. 2016). PCORI has sought to improve the rigor of research methods application in general by developing methodology standards, which are publicly available here (Committee 2017).

AHRQ convened a technical expert panel of health services researchers in late 2016 to develop Learning Health System Researcher Core Competencies (Forrest et al. 2018). The resulting work yielded seven Learning Health System competencies including: Systems Science, Research Questions and Standards of Scientific Evidence, Research Methods, Informatics, Ethics of Research and Implementation in Health Systems, Improvement and Implementation Science; and Engagement, Leadership, and Research Management. Four of these seven competencies—Research Questions and Standards of Scientific Evidence, Research Methods, Ethics of Research, and Engagement, Leadership, and Research Management—map directly to the current V2.1 HSR competencies listed in Table 1. The three unique Learning Health System competencies from the technical expert panel—Systems Science, Informatics, and Improvement and Implementation Science—are domains of other HSR competencies reported herein but are not elevated to their own competencies in V2.1. HSR trainees, doctoral programs, and practitioners may want to identify training opportunities to master these competencies, given that they are anticipated to increase in importance in the coming decade.

### Modalities for Delivering Competencies

The modalities for delivering content designed to develop the HSR competencies have primarily been via didactic and experiential learning (see Table 2). Didactic learning to teach skills and methods of each core competency has been delivered through a range of venues, including traditional coursework, Internet modules, intensive workshops, summer institutes, or mock study sections. There are emerging training modalities, such as “flip the classroom,” that may be useful to integrate into doctoral programs, as public health students have reported satisfaction with this approach in two studies (Galway et al. 2014; Moraros et al. 2015).

**Table 2:** Delivery of Health Services Research Doctoral Core Competencies

Delivery Type	Examples
Didactic learning	Prerequisite readings before matriculation Courses (single instructor and team instruction) Semester and quarter long courses Modular or short courses Internet modules Faculty‐ or student‐led seminars Workshops Journal clubs Summer institutes Mock study sections
Experiential learning	Research assistantships Teaching assistantships Faculty and peer mentoring Applied internships/practicum experiences, on‐the‐job training, shadowing Student‐ or faculty‐led consulting Peer advising Working with multi‐disciplinary research teams Networking with visiting scholars Dissertation and grant proposal writing, submission, and revision Conference participation Oral dissemination opportunities (posters, conference presentations, at regional and national meetings) Writing journal articles, developing publication strategies, responding to reviewers’ comments Writing policy briefs (testimony briefs, press releases) Policy development Stakeholder and community collaboration Developing and implementing intervention programs Job talks with cognitive debriefing

The modalities of experiential learning include research and teaching assistantships, faculty and peer mentoring programs, and formal opportunities to present one's work at poster sessions, regional and national meetings, stakeholder meetings, and other venues. An important type of experiential learning that is becoming increasingly important is the conduct of research in partnership with an external health care organization or as an intern or employee doing intramural research within a health care organization. Through active engagement in these experiential learning activities, HSR trainees gain important experience in the conduct and dissemination of HSR. Learners also gain an appreciation for the practical and logistical challenges of converting novel ideas into actionable research and communicating results in ways that resonate with each stakeholder group.

### Competency Assessment

To evaluate the delivery of content designed to develop HSR competencies, there is a need to assess HSR doctoral students’ and postdoctoral trainees’ mastery of competencies. An array of assessment tools, presented from various vantage points regarding who is conducting the assessment (faculty, students, alumni, employers, or other outside evaluators) and who is undergoing assessment appears in Table 3. The most common assessments used in most training programs are writing assignments in courses, course grades, and qualifying examinations. These assessment approaches may need to be revisited as new training modalities evolve in the coming years.

**Table 3:** Methods for Assessing Health Services Research Doctoral Core Competencies

Who Is Doing the Assessment?	Who/What Is Being Assessed?	Assessment Opportunities
Faculty	HSR program	Peer course evaluations Curriculum review (program, department, school) Crosswalk between competencies and learning objectives
	HSR students	Course grades Faculty written assessments Faculty oral assessments Progress reports/plans Benchmarking against other students RA and TA evaluations Critique of independent study progress Critique of research papers Qualifying/area exams Dissertation proposal defense Dissertation defense Exit interviews
Students	HSR program	Student course and seminar evaluations Graduate job placement Peer‐reviewed publications Presentations Posters News and op‐ed articles Gray literature Research funding Exit interviews
	Self‐ and peer‐assessment	Self‐assessments Mock study section Peer reviews of work in progress Community service
Alumni	HSR program	Alumni surveys (1 year, 3–5 year, 5+ years) Peer collaboration network Continuing education
	Self‐ and peer‐assessment	Job offers Job history: leadership positions, impact on policy and practice Peer‐reviewed publications Presentations Posters News and op‐ed articles Gray literature Grant funding
Employers	HSR program	Hiring of graduates with HSR degrees Job offers and placements for HSR graduates
	HSR students	Internship/practicum evaluation
	HSR employees	Employee performance evaluation
Other outside evaluators	HSR program	Study section reviews of training grants Stakeholder reviews University reviews CEPH reviews External advisory board reviews
	HSR students	Study section scores and reviews of student submitted grants Patient reviews of clinical work Community reviews of student interventions Critique of journal article submissions

### Training Programs and HSR Typology

The core competencies presented above include an expansive list of professional skills that no one scholar is likely to possess. Instead, the list is more reflective of the collection of competencies that a team of health services researchers may collectively comprise. Anecdotally, we believe that a finite number of different health services researcher “types” exist. Each of these “types” of health services researcher may be approximately characterized by the questions asked, methods and theories used, and perhaps the journals where their work appears (Table 4). We have observed that employers (including universities) that recruit health service researchers are typically looking for a specific type in a given search (e.g., cancer health services researcher or implementation scientist with training in organizational behavior).

**Table 4:** Typology of Health Services Researchers

HSR Type	Brief Description	Sample of Questions Asked	Decision Maker or Unit of Analysis	Typical Methods	Journals Published
Quantitative and economic methods health services researchers	Questions framed from perspective of public policy stakeholders	What is the effect of program/policy X on outcome Y?	Patient, clinician, or organization	Causal inference study designs, comparative effectiveness approaches using governmental health surveys, claims data, and other longitudinal data	Policy and economic journals (e.g., Health Affairs, Journal of Health Economics)
Clinical health services researchers	Questions framed from the perspective and experiences of a defined patient population with a common disease or condition	How do patients with disease or condition X fare with respect to intervention Y? Questions frequently focus on cost, quality, or access to services	Patient or clinician	Epidemiological and economic study designs	Clinical journals (e.g., New England Journal of Medicine)
Organizational health services researchers	Questions framed from the perspective of health delivery organizations leaders	How is organizational issue X (e.g., EHR adoption, nurse shortage) associated with some financial, quality, human resource, or other organizational outcome of interest?	Clinician or organization (e.g., hospital, nursing home)	Quantitative, qualitative, and mixed methods	Health care management journals (e.g., Health Care Management Review)
Social/behavioral sciences health services researchers	Advances in this area help scientifically explore patient experiences with the health care system	What can be learned by understanding the experiences of patients and/or others that may lead to improvements in health and health care delivery?	Patient	Qualitative and mixed‐methods approaches are common	Social science and quality journals (e.g., Social Science in Medicine, American Journal of Medical Quality)
Public health services researchers	Researchers often explore the association between social determinants of health and some aspect of public health system performance	What is the impact of intervention X on community health outcomes? In what ways can our public health system be improved?	Patient or community	A wide range of quantitative and qualitative approaches to analyze data collected by NACCHO, ASTHO, CDC, and others	Public health journals (e.g., American Journal of Public Health)

Many HSR training programs typically specialize in the training of individuals with expertise in one or more of these categories, so this typology may assist training programs in selecting the combination of competencies, modalities, and assessments that best prepare students for the target market that their programs focus upon. Likewise, the typology may assist current and future doctoral students and employers to better articulate their needs and wants from an individual who identifies themselves as a given type of health services researcher. Importantly, many health services researchers develop skills, interests, and expertise in one or more of these groupings throughout their training and career.

## Discussion

Core competencies in HSR need to be seen as a continually evolving work in progress because new research questions arise, new methods are developed, and the trans‐disciplinary nature of the field leads to new multidisciplinary and team building needs. It is not intended that these competencies ever be used for accreditation of training programs. Instead, the competencies are offered as suggestions to training programs who can innovate on ways to advance how the field trains future health services researchers. Nevertheless, we believe that the 11 core competencies are standing the test of time and still represent a core that defines what HSR researchers need to master in order to address the complexities of microsystem to macro‐system research that HSR entails. The importance of building effective research teams that span the methods required to answer particular questions effectively is a growing success factor for the field.

The depth and breadth of qualitative and quantitative methods that are worthy of mastery are daunting, but few (if any) health services researchers master all of them. While successful researchers may not need to master all of the methods, we believe familiarity with a wider array of methods and ability to collaborate with a team representing a range of disciplines and methodologies is an extremely useful competency. In many cases, this involves learning how to translate between research vernaculars where different intellectual traditions use different terminology to describe the same concepts and methods (Maciejewski, Weaver, and Hebert 2011).

Academic programs should develop their own approaches to training students in these competencies that fit into the contexts and histories of the structure of their particular programs. For researchers engaged in lifelong learning, these core competencies may be a useful template for establishing individual goals and practices that improve their capabilities and ability to form and lead research teams. Building these research teams could explicitly use the competencies and/or the typology as a template for making sure appropriate skillsets are present on their teams. Entities promoting learning organization goals also may find these Core Competencies useful in setting and measuring outcomes for their individuals and teams conducting HSR.

There are two important issues that we did not grapple with here that would merit consideration in the future. First, the availability of new modalities of competency delivery (e.g., flipped classrooms, asynchronous modalities to come) may prove to be more effective for teachers and students and may enable greater depth and breadth of content delivery. However, there is little evidence to support this supposition or the conditions under which these modalities realize optimal student outcomes. More broadly, the principles used to conduct HSR could be applied to determining the optimal matching of modality and subject/content. We recommend that the field conduct formal evaluation when these newer modalities are tested to inform how best to employ them. We further recommend that AcademyHealth and journals publishing HSR content should consider creating venues to disseminate the results of such evaluations. Second, there may be value in integrating Learning Health System Researcher Core Competencies (e.g., informatics, implementation science) more fully into HSR doctoral training programs (Forrest et al. 2018). A considered deliberation about the value of elevating tenets from informatics and implementation science to the HSR competencies would be warranted in the future.

To be a useful guide to doctoral and training programs, students, and employers, core competencies must evolve over time as research questions, types of HSR practitioners, methods, and data change. The current modest revision to the V2 core competencies and formal presentation of content delivery and assessment methods developed in the V2 conference represents the latest iteration for what must be an ongoing dialogue about the training that HSR practitioners should receive. Future revision of these competencies would benefit from multidisciplinary collaboration after review of related competencies and literature as was done to develop the V1 competencies. Through mastery of an increasing number of these competencies, our field will be better prepared to address the pressing challenges facing the health care system using appropriate methods and high‐quality data.

## Supporting information

Appendix SA1: Author Matrix.
